# The impact of long-term care insurance on the subjective well-being of middle-aged and older individuals in rural China an empirical analysis based on CHARLS data

**DOI:** 10.3389/fpubh.2025.1662574

**Published:** 2025-10-08

**Authors:** Zhenwei Liu, Yinan Yang, Guoli Mo, Chunzhi Tan, Weiguo Zhang, Wei Jia

**Affiliations:** ^1^China-ASEAN School of Economics, Guangxi University, Nanning, China; ^2^School of Public Administration, Hunan University, Changsha, China; ^3^College of Management, Shenzhen University, Shenzhen, China

**Keywords:** long-term care insurance, middle-aged and older individuals, rural areas, subjective well-being, difference-in-differences, mental health

## Abstract

The severe, often overlooked, mental health crisis among the aging population in rural China poses a pressing social challenge. This study investigates the role of a major policy intervention, Long-Term Care Insurance (LTCI), in enhancing their subjective well-being (SWB). Using panel data from the China Health and Retirement Longitudinal Study (CHARLS) for 2011–2020 and a multi-period difference-in-differences (DID) model, we find that LTCI implementation significantly improves the SWB of rural middle-aged and older residents. The underlying mechanisms include alleviated medical expenditure burdens, improved health status, and increased consumption. Crucially, our findings reveal a powerful equity-enhancing effect: the well-being benefits are substantially greater for the most vulnerable individuals—those with lower income, poorer health, and limited education. This study highlights LTCI’s vital function not only as a financial safety net but also as a crucial tool for promoting mental wellness and social equity in rural China. Policy should prioritize the expansion and optimization of LTCI to better support this at-risk demographic.

## 1 Introduction

In the vast rural landscapes of China, a silent mental health crisis is intensifying. According to sampling data from China’s National Health Commission’s 2024 “Development of Aging Undertakings Bulletin,” the rural population aged 60 and over has reached 140 million, accounting for 23.7% of the total rural household registration population and exceeding a staggering 28% of the resident population. This demographic pressure exacerbates an escalating risk of disability within a fragile care system. Compounding this, the “China Aging Development Report 2024” Blue Book, released in December 2024, reveals that 26.4% of the nation’s older adults suffer from symptoms of depression. For the vulnerable rural older adults, already caught in a state of eroding physical health and dwindling traditional support from “hollowed-out” families, functional limitation acts as a powerful catalyst, transforming latent psychological distress into a severe crisis marked by deep feelings of loneliness, depression, and fear.

In response to this pressing challenge, China has vigorously promoted Long-Term Care Insurance (LTCI) as the nation’s “sixth pillar” of social insurance. However, existing scholarship suffers from a significant urban-centric bias. This scholarly gap reflects an initial bias in the policy’s design itself; many LTCI pilots prioritized participants of urban employee medical insurance, while rural residents covered by different schemes were often included later or not at all. Consequently, when rural populations are considered in research, it is typically as a brief point of comparison from a broader urban or national perspective, rather than as the focus of a comprehensive, systematic investigation. This scholarly neglect is particularly pressing given that the rural older adults constitute a population with distinct vulnerabilities and urgent care needs, making them a crucial subject for dedicated inquiry. This study’s primary contribution, therefore, is to address this critical gap by providing a systematic, in-depth empirical analysis focused exclusively on how LTCI causally impacts the mental health and life satisfaction of this overlooked population. Our central research question is: Does the implementation of LTCI affect the subjective well-being (SWB) of China’s vulnerable rural middle-aged and older population, and if so, through what mechanisms does this influence operate?

Building on this core innovation, this study makes two further contributions. Second, by concentrating on disadvantaged rural populations, it illuminates the differentiated welfare effects of social security policy within China’s persistent urban–rural dual structure. Third, it provides actionable policy implications for other developing countries grappling with population aging, particularly concerning the tailoring of long-term care systems to rural needs and bridging the protection gap left by the erosion of traditional family support.

To empirically answer our research question, this study utilizes five waves of panel data (2011-2020) from the China Health and Retirement Longitudinal Study (CHARLS), a nationally representative survey organized by the National School of Development at Peking University. Employing a multiple time-point difference-in-differences (DID) model, our analysis reveals that LTCI significantly enhances the SWB of rural middle-aged and older residents. The mechanisms driving this effect are the alleviation of medical financial burdens, the improvement of physical health, and an enhancement in consumption levels. Heterogeneity analyses further show that the policy’s positive effects are more pronounced for individuals with lower incomes, poorer health, and less education, underscoring LTCI’s vital function as a tool for promoting both mental wellness and social equity in rural China.

## 2 Policy context and research progress

### 2.1 Institutional background

LTCI provides financial support to insured individuals who require assistance with activities of daily living (ADL) due to functional limitations or frailty. It covers long-term medical and custodial care in various settings, including nursing homes, medical institutions, and private residences. In China, LTCI is positioned as the “sixth pillar” of social insurance, supplementing the traditional pension, medical, work-related injury, unemployment, and maternity insurance systems. Funded through a mutual assistance model, China’s LTCI was initially designed for the older adults and severely disabled, with its coverage and service scope gradually expanding over time.

LTCI originated in the mid-to-late 20th century. The Netherlands pioneered mandatory social LTCI in 1968 with its Exceptional Medical Expenses Act. The United States followed by introducing private LTCI in 1975, while the United Kingdom launched its long-term care allowance program. Subsequently, Germany institutionalized its LTCI in 1994, and Japan established its Nursing Care Insurance in April 2000. In China, the development of LTCI began with a pilot program in Qingdao in 2012, which provided crucial implementation experience. These efforts were expanded in 2016 when pilots were launched in 15 Chinese cities, targeting enrolled middle-aged and older individuals with prolonged disabilities. The primary aim was to provide basic living assistance and alleviate the medical expenditures for severely disabled persons. To further accelerate the national rollout, in September 2020, joint guidelines from China’s National Healthcare Security Administration and Ministry of Finance expanded the pilot programs to 49 cities. As of January 2025, data from the Chinese National Healthcare Security Administration show that LTCI covers 180 million people, with over 2.6 million disabled insured individuals receiving benefits and fund expenditures exceeding 80 billion yuan.

A unified national LTCI system in China is an inevitable trend. This vital social security tool integrates care resources via risk-pooling, improving older adults quality of life, easing family care burdens, and fostering socio-economic stability. Its design balances equity and sustainability, systematically addressing population aging and disability challenges.

### 2.2 Research progress on the policy effects of LTCI

The literature on the effects of LTCI policy is multidimensional, with research spanning health outcomes, family caregiving dynamics, and health equity.

Regarding health outcomes, studies using difference-in-differences (DID) methodologies indicate that LTCI significantly reduces older adult disability, hospitalization costs, and overall medical expenditures (1–6). The underlying mechanisms include the substitution of inpatient services, fewer medical visits, and improved health behaviors (5, 7). These effects are often more pronounced for individuals with chronic diseases and for the oldest-old (8).

In the context of family caregiving, while some studies suggest LTCI may “crowd-in” family care, a broader consensus shows that it significantly eases the burden on informal caregivers (1, 9–11). It has also been found to improve the physical and mental health of caregivers and to shift household spending from precautionary savings to non-medical consumption (8, 12, 39).

From a health equity perspective, the findings are more complex. LTCI generally improves health welfare but can also worsen health outcome inequalities between urban and rural areas and across income groups (13, 14). Disparities in access to formal care are also evident, with women, individuals living alone, and urban residents tending to benefit more (1, 15). China’s LTCI pilots have revealed challenges such as policy fragmentation and insufficient community and home-based care. Scholars recommend that improving equity requires expanded coverage, differentiated benefits, and stronger resource integration (2, 13, 15).

Despite substantial multidimensional knowledge accumulation, structural limitations hinder academic consensus and theoretical depth. Existing research predominantly uses an “objective utility” framework, focusing on explicit indicators like healthcare cost control, care service accessibility, and family caregiving substitution effects. However, the psychological well-being of insured individuals remains underexplored theoretically and empirically. This orientation yields a tool-focused evaluation system, with gaps in longitudinal tracking and dynamic analysis of key older adult psychological indicators (e.g., SWB, perceived dignity, life satisfaction). Furthermore, studies often lack inclusive sample designs, neglecting policy effects on disadvantaged rural middle-aged and older groups—critical LTCI beneficiaries. Limited empirical exploration of their policy experiences and welfare acquisition constrains policy evaluation ecological validity, evidence-based policymaking precision, and institutional optimization direction.

## 3 Theoretical framework and research hypotheses

As a critical component of social care for the older adults, LTCI significantly impacts rural areas by providing essential medical and nursing support. Its implementation can generate positive externalities that influence the economic conditions, health, and quality of life for middle-aged and older individuals in these communities. However, the unique characteristics of rural settings introduce complexity. While LTCI has the potential to enhance well-being, implementation challenges could inadvertently exacerbate financial burdens and diminish its intended benefits.

### 3.1 Medical burden

LTCI is expected to alleviate the medical burden for rural middle-aged and older individuals through several mechanisms. First, by providing professional medical and nursing services, it can reduce outpatient visits and hospitalizations while partially reimbursing expenses, thereby lowering direct healthcare costs (2–6). Second, consistent with human capital theory, LTCI can enhance the professionalism and quality of care providers, which in turn reduces unnecessary medical expenditures (16). Third, drawing from health promotion theory, LTCI may improve the overall health of its rural beneficiaries, consequently diminishing their demand for medical services (8). The link between reduced financial strain and improved well-being is well-supported in the literature. According to stress-adaptation theory, alleviating the medical burden mitigates economic stress, which reduces psychological strain and enhances SWB. Empirical research confirms this; for instance, household debt has been shown to diminish SWB, while relieving medical expenditure burdens directly elevates it and protects mental health (17–19).

Conversely, the implementation of LTCI in rural areas may inadvertently increase the medical burden on middle-aged and older individuals for several reasons. First, enrollment in LTCI often requires prior participation in other medical insurance plans (such as those for urban employees or urban–rural residents). Although the contributions are modest, these premiums can represent a considerable financial burden for rural older residents who lack a stable income. Second, because LTCI is an insurance mechanism without guaranteed returns, premiums paid by rural individuals who never become eligible for claims constitute a net financial loss, effectively increasing their healthcare-related costs. Third, according to moral hazard theory, the sense of security afforded by LTCI coverage might reduce an individual’s vigilance regarding their own health, potentially leading to a decline in their health status and higher long-term medical expenditures.

In summary, LTCI implementation effects in rural areas are complex. This study thus proposes two competing hypotheses:

*Hypothesis 1a*: LTCI enhances the SWB of rural middle-aged and older individuals by alleviating their medical burdens.

*Hypothesis 1b*: LTCI reduces the SWB of rural middle-aged and older individuals by increasing their medical burdens.

### 3.2 Health status

Beyond its impact on household expenditures, LTCI also influences the SWB of rural middle-aged and older individuals by improving their health (14). First, according to health capital theory, LTCI enhances health by providing access to professional caregiving and medical services. This is particularly impactful in rural areas with scarce medical resources, where professional caregivers can help address physical ailments, facilitate recovery, and strengthen physical resilience. Second, drawing on health promotion theory, LTCI can foster greater health awareness. In rural settings, caregivers often act as health educators, empowering middle-aged and older individuals with improved health literacy and preventive capabilities. Empirical studies confirm these pathways, showing that long-term care services significantly enhance the physical health of insured individuals (5, 8).

A growing body of literature indicates a significant link between the health of middle-aged and older adults in rural areas and their SWB (20–22). First, improved health allows the rural older adults to manage their self-care more effectively, which reduces dependency on their children. This, in turn, fosters a greater sense of autonomy and self-esteem, thereby improving SWB. Second, individuals who do not face physical barriers in their daily lives are better able to socialize and participate in community organizations. This activity strengthens their social connections and support systems, fostering a higher level of SWB. Finally, rural individuals who are in good physical and mental health are more inclined to seek self-fulfillment by engaging in volunteer work or community service. Such activities cultivate a profound sense of personal worth and achievement, ultimately leading to enhanced SWB.

This study thus proposes the following hypothesis:

*Hypothesis 2*: LTCI enhances the SWB of rural middle-aged and older individuals by improving their health status.

### 3.3 Consumption level

As a form of social security, LTCI significantly reduces the financial burden on rural households by sharing the care costs for disabled individuals. In resource-scarce rural areas, these reduced expenditures free up disposable income, thereby enhancing consumption capacity (8, 23, 24). LTCI also improves access to formal caregiving services, which can prevent family members from having to withdraw from the labor market, thus strengthening household economic stability. This alleviation of financial pressure and the resulting increase in consumption capacity create new opportunities for rural middle-aged and older individuals, particularly for spending on healthcare, personal services, and cultural and recreational activities.

**Figure 1:**
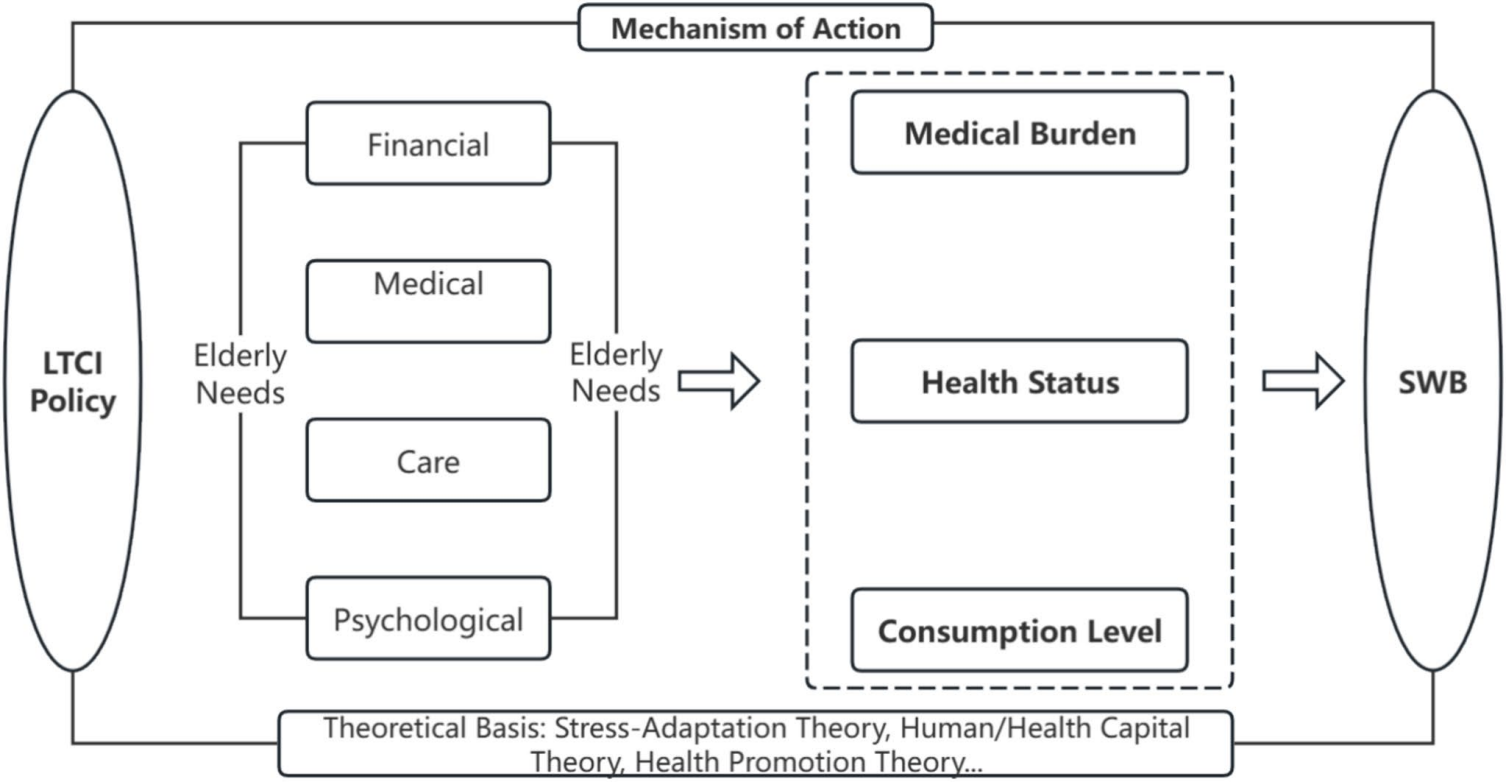
Mechanism of action.

Classical economic theories help explain why enhanced consumption positively impacts SWB. Maslow’s hierarchy of needs, for example, suggests that consumption not only fulfills basic physiological and safety needs but also provides a pathway to satisfying higher-level needs such as belongingness, esteem, and self-actualization (25). The consumption capacity unlocked by LTCI can first improve the satisfaction of basic living requirements for rural middle-aged and older individuals and then promote psychological well-being through spending on cultural and social activities. Furthermore, while the law of diminishing marginal utility implies that consuming more of the same good yields progressively smaller gains, a shift in the structure of consumption—from basic necessities toward higher-level cultural and healthcare goods—can produce a more significant enhancement in SWB. Substantial literature confirms this positive link between increased and diversified consumption and improved well-being (26–28) (Figure 1).

This study thus proposes the following hypothesis:

*Hypothesis 3*: LTCI enhances the SWB of rural middle-aged and older individuals by facilitating increased consumption.

## 4 Research design

### 4.1 Model specification

#### 4.1.1 Baseline regression

This study employs a difference-in-differences (DID) model to estimate the impact of LTCI on the well-being of rural middle-aged and older individuals. This approach is specifically chosen to account for the staggered timing of the policy’s implementation across various pilot cities. The model is specified in Equation 1.


(1) $$\mathrm{SW}B_{\mathrm{ijt}}=\beta_{0}+\beta_{1}(\text{Trea}t_{\mathrm{it}}\times {\text{Time}}_{t})+\gamma X_{\mathrm{ijt}}+\mu_{j}+\theta_{t}+\varepsilon_{\mathrm{ijt}}$$

In Equation 1, subscripts *i*, *j*, and *t* denote city, individual, and time, respectively. Treat*_ij_* is a dummy for individual *j* in pilot city *i* (1 = treatment, 0 = control). Time*_t_* is a dummy for the post-pilot period (1 = post-pilot, 0 = pre-pilot). The interaction Treat*_ij_* × Time*_t_* is the core explanatory variable for LTCI. X*_ijt_* are control variables, μ*_j_* and θ*_t_* are individual and time fixed effects, and $\varepsilon_{\mathrm{ijt}}$ is the random error. Standard errors are city-level clustered.

#### 4.1.2 Parallel trend test

The validity of the DID model hinges on the parallel trend assumption. This assumption posits that, in the absence of the policy, the average well-being of rural middle-aged and older individuals would have followed similar trends in both the treatment and control groups. To empirically test this prerequisite, we adopt an event-study approach following Liu et al. (8). This involves augmenting the baseline model with interaction terms between the treatment group indicator and time dummies for the years before and after the LTCI intervention, as specified in Equation 2.


(2) $$\begin{array}{l} \mathrm{SW}B_{\mathrm{it}}=\beta_{0}+\sum_{n=1}^{5}y_{n}\mathrm{pr}e_{\mathrm{it}}^{n}+\beta_{1}\text{curren}t_{\mathrm{it}}\\ +\sum_{n=1}^{5}\delta_{n}\mathrm{pos}t_{\mathrm{it}}^{n}+\sum \gamma_{i}\text{Contro}l_{\mathrm{it}}+u_{j}+\theta_{t}+\varepsilon_{\mathrm{it}} \end{array}$$

*Pre_n_* is a dummy for n years before LTCI pilot implementation, Current for the implementation year, and *Post_n_* for n years after implementation. Significant positive coefficients for pre-policy terms (*Pre_n_*) would suggest potential estimation bias from unobserved confounders. Conversely, if these coefficients are not statistically significant, it lends support to the causal interpretation that any observed improvement in the well-being of rural middle-aged and older individuals can be attributed to the LTCI policy.

### 4.2 Variable definitions and measurements

#### 4.2.1 Dependent variable

SWB is measured via three dimensions: life satisfaction, positive affect, and negative affect. Life satisfaction is from “Are you satisfied with your life?.” Positive affect uses “Feeling hopeful about the future” and “Feeling happy”; negative affect uses six items: “Feeling bothered by minor things,” “depressed,” “everything an effort,” “fearful,” “lonely,” and “unable to continue with life.” SWB is calculated as: Positive Affect + Life Satisfaction – Negative Affect (range: −50 to 42; higher scores = greater well-being).

This composite measure is theoretically grounded in the classic tripartite model of SWB, which posits that SWB comprises a cognitive component (life satisfaction) and two affective components (positive and negative affect) (29). This framework remains the methodological standard in the field and continues to be employed in recent, high-impact research (30, 31). Therefore, constructing our dependent variable based on this well-established approach ensures both theoretical coherence and alignment with current best practices.

#### 4.2.2 Core explanatory variable

The core explanatory variable is a dummy variable, LTCI, which indicates whether a middle-aged or older individual is exposed to the LTCI policy. To mitigate potential endogeneity, this variable is defined based on the policy’s implementation timing rather than individual enrollment. Specifically, respondents residing in cities where LTCI has been implemented and who are covered by basic employee or resident medical insurance are assigned to the treatment group (coded as 1), while all others are assigned to the control group (coded as 0). The treatment group comprises individuals from 22 pilot cities, with the control group drawn from non-pilot cities.

#### 4.2.3 Control variables

Following previous studies (6, 8), the control variables include several categories: individual characteristics (gender, age, education, marital status, household registration, social activities, retirement); health behaviors (exercise, smoking and drinking, sleep); household attributes (total income, size); social support (medical and pension insurance participation); and regional factors (GDP per capita, population aging, and the number of healthcare institutions, beds, and personnel). Detailed definitions and descriptive statistics for these variables are presented in Table 1.

**Table 1:** Measurement of key variables.

Variable type	Variable name	Variable symbol	Definition
Dependent variable	Subjective Well-Being	SWB	SWB is calculated as the sum of positive affect and life satisfaction minus negative affect, with a value range of [−50, 42]; higher values indicate greater well-being.
Core independent variable	Long-Term Care Insurance	LTCI	Equals 1 if the respondent resides in a pilot city and belongs to the covered group; equals 0 if the city is not piloting LTCI or if the respondent is not covered.
Individual characteristics	Gender	gender	Male = 1; Female = 0
	Age	age	Logarithm of respondent’s age
	Education	edu	Education level, ranging from 1 to 4; higher values indicate higher education attainment.
	Marital Status	marry	Married = 1; Others = 0
	Social Activities	social	Participated in club activities, playing mahjong, chess, cards, or community activities in the past month = 1; otherwise = 0
	Retirement Status	retire	Retired = 1; Not retired = 0
Health characteristics	Exercise	exercise	Exercises = 1; No exercise = 0
	Unhealthy Behaviors	drink_smoke	Smoking or drinking behavior = 1; No unhealthy behavior = 0
	Sleep Duration	sleep	Logarithm of sleep duration
Household characteristics	Total Household Income	Incomel_total	Logarithm of total household income
	Household Size	Family_size	Number of household members
Intergenerational support	Financial Support from Children	fcamt	Logarithm of the amount of financial support received from children
Social support	Medical Insurance	ins	Enrolled in medical insurance = 1; Not enrolled = 0
	Pension Insurance	pension	Enrolled in pension insurance = 1; Not enrolled = 0
Regional characteristics	Number of Health Institutions	health_inst	Logarithm of the number of health institutions
	Number of Hospitals	hospitals	Logarithm of the number of hospitals and health centers
	Number of Hospital Beds	beds	Logarithm of the number of hospital and health center beds
	*Per Capita* GDP	gdp	Logarithm of per capita GDP
	Aging Degree	old	Proportion of population aged 65 and above
Mechanism test	Medical Burden 1	MB1	Continuous variable, logarithm of annual out-of-pocket inpatient expenses
	Medical Burden 2	MB2	Continuous variable, logarithm of out-of-pocket outpatient expenses
	Medical Burden 3	MB3	Number of hospitalizations
	Medical Burden 4	MB4	Number of outpatient visits
	Health Status 1	H1	Composite score based on difficulty in completing daily activities (housework, cooking, shopping, financial management); range 0–6, with lower values indicating better health.
	Health Status 2	H2	Discrete variable based on self-rated health status; range 1–5, with higher values indicating better health.
	Health Status 3	H3	Diagnosed with any of 14 chronic diseases such as hypertension or diabetes = 1; otherwise = 0
	Total Consumption	TC	Logarithm of total consumption expenditure
	Enjoyable consumption	EC	Logarithm of expenditure on enjoyment-related consumption

### 4.3 Data description and descriptive statistics

#### 4.3.1 Data description

This study uses empirical data from the China Health and Retirement Longitudinal Study (CHARLS), organized by Peking University’s National School of Development. The dataset includes five waves (2011, 2013, 2015, 2018, 2020), covering ~17,000 respondents in 150 counties and 450 villages. Focusing on Chinese households with members aged 45+, CHARLS offers rich micro-data on demographics, assets, family structure, health, medical insurance, etc., supporting China’s population aging research.

The final analytical sample was constructed through a multi-step data processing procedure: (1) the sample was restricted to rural individuals aged 45 and over, with observations having significant missing values on key variables removed; (2) some cities were excluded to ensure the integrity of the treatment and control groups due to data limitations or confounding local policies;^1^ (3) policy implementation timing and coverage groups were defined as specified in Table 2; and (4) minor missing values in control variables were handled using linear interpolation, and all continuous variables were winsorized at the 1% level to mitigate the influence of outliers. This process yielded a final panel dataset of 47,330 valid samples across five periods.

**Table 2:** Implementation of LTCI policies in 22 selected pilot cities.

No.	City	Year of policy implementation	Covered population
1	Qingdao	2012	b (urban areas)
		2015	b (rural areas)
2	Weifang	2015	a
		2024	b
3	Shangrao	2017	a
		2019	b
4	Jinan	2016	a
		2021	b
5	Jingmen	2016	a
		2018	b
6	Chengde	2017	a
7	Anqing	2017	a
		2026	b
8	Chengdu	2017	a
		2021	b
9	Xuzhou	2017	a
		2018	b
10	Guangzhou	2017	a
		2021	b
11	Linyi	2017	a
		2023	b
12	Qiqihar	2017	a
13	Suzhou	2017	b
14	Liaocheng	2015	a
		2023	b
15	Ningbo	2017	a
		2023	b
16	Chongqing	2019	a
17	Binzhou	2017	a
		2023	b
18	Zaozhuang	2019	a
		2023	b
19	Weihai	2018	a
		2019	b
20	Dezhou	2019	a
		2023	b
21	Tianjin	2020	a
		2026	b
22	Kunming	2020	a
		2025	b

‘a’ denotes urban employee medical insurance participants; ‘b’ denotes urban employee and resident medical insurance participants. Post-2020 implementation dates are included as these cities serve as a specific control group in the “Refining the Control Group” robustness check (Section 5.4.4).

**Figure 2:**
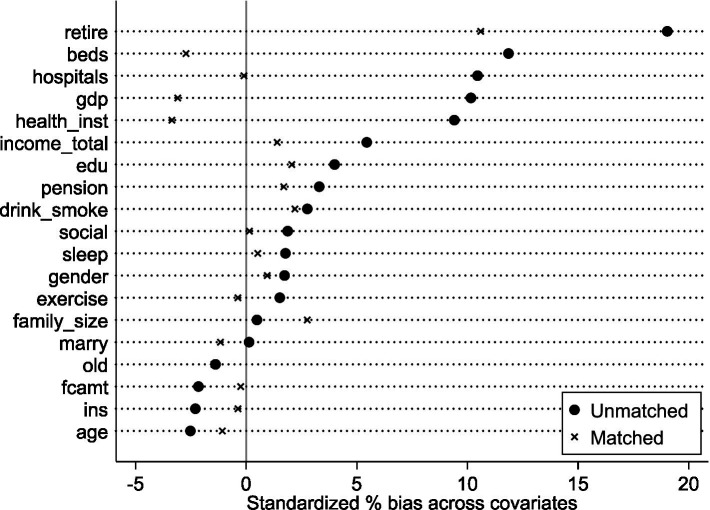
Balance test. Nearest-neighbor matching within caliper (1:4 ratio, caliper = 0.05).

However, a substantial treatment-control imbalance existed (treatment group: 3.03% of total sample). To enhance DID comparability, maintain sample size, and mitigate endogeneity from systematic differences, propensity score matching (PSM) used a nearest-neighbor 1:4 strategy. Robustness checks used 1:3, 1:2, and 1:1 nearest-neighbor matching. Post-matching balance tests (Figure 2) show most covariates had no significant differences, confirming successful balance. This resulted in 5,849 valid observations (1,432 treatment, 4,417 control), partially alleviating systematic bias concerns. Finally, multicollinearity tests showed all VIFs < 5.61 (average VIF = 1.81), indicating no significant multicollinearity.

#### 4.3.2 Descriptive statistics

Table 3 presents the descriptive statistics for key variables, categorized by the total sample, as well as by the treatment and control groups. On average, individuals in the treatment group report higher levels of SWB and greater economic security—as indicated by total household income, consumption, and coverage by medical and pension insurance—compared to those in the control group. This initial comparison suggests that LTCI may positively influence well-being and economic conditions. However, the descriptive data also reveal that the treatment group has a slightly lower mean health status. This implies that the direct health effects of LTCI may be limited or perhaps require a longer period to manifest. Additionally, the demographic differences between the groups in terms of gender, age, and education are small, suggesting that the treatment and control groups are largely comparable on these observable characteristics. In summary, these preliminary findings suggest that while LTCI shows potential for enhancing well-being and economic security, its effects on health appear more complex, warranting the rigorous econometric analysis that follows.

**Table 3:** Descriptive statistics.

Variable	Full Sample		Treatment Group		Control Group	
	Mean	SD	Mean	SD	Mean	SD
SWB	−1.1010	5.1034	−0.4365	4.7398	−1.3165	5.1982
Treated	0.2448	0.4300	1.0000	0.0000	0.0000	0.0000
Gender	0.4921	0.5000	0.4986	0.5002	0.4899	0.5000
Age	4.1115	0.1501	4.1086	0.1570	4.1124	0.1478
Edu	1.9417	0.9542	1.9707	0.9708	1.9323	0.9487
Marry	0.8908	0.3120	0.8911	0.3117	0.8907	0.3121
Social	0.5090	0.5000	0.5161	0.4999	0.5067	0.5000
Retire	0.1151	0.3191	0.1634	0.3699	0.0994	0.2992
Exercise	0.8938	0.3081	0.8974	0.3036	0.8927	0.3096
Drink_smoke	0.5784	0.4939	0.5887	0.4922	0.5751	0.4944
Sleep	1.9576	0.2912	1.9615	0.2868	1.9563	0.2926
income_total	15.7267	0.0271	15.7277	0.0157	15.7264	0.0299
Family_size	2.8805	1.3705	2.8855	1.3258	2.8789	1.3848
Fcamt	5.3756	3.8764	5.3127	3.8496	5.3961	3.8853
Ins	0.9325	0.2510	0.9281	0.2585	0.9339	0.2485
Pension	0.6579	0.4745	0.6697	0.4705	0.6541	0.4757
Health_inst	8.5992	0.9417	8.6658	0.9292	8.5776	0.9449
Hospitals	5.3598	0.7442	5.4187	0.7515	5.3407	0.7409
Beds	10.5082	0.6911	10.5691	0.6581	10.4885	0.7005
Gdp	11.1626	0.4897	11.1995	0.4662	11.1506	0.4966
Old	12.7621	2.9303	12.7341	2.0938	12.7712	3.1545
MB1	0.3512	1.4289	0.2318	0.7820	0.3899	1.5810
MB2	0.2108	0.7590	0.1725	0.6520	0.2232	0.7903
MB3	0.6024	2.1518	0.5704	2.0886	0.6128	2.1720
MB4	0.5583	1.7393	0.5568	1.7106	0.5588	1.7486
H1	0.7295	0.4442	0.6634	0.4727	0.7510	0.4325
H2	0.3083	0.9053	0.2400	0.7970	0.3298	0.9359
H3	3.1892	1.0223	3.3275	1.0363	3.1468	1.0143
TC	12.9231	0.1963	12.9403	0.1090	12.9179	0.2156
EC	11.9172	0.1469	11.9368	0.1576	11.9110	0.1428
N	5,849		1,432		4,417	

## 5 Empirical results

### 5.1 Baseline regression analysis

Table 4 presents the baseline regression results for the impact of LTCI on the SWB of rural middle-aged and older individuals. The specifications, presented in columns (1) through (5), sequentially introduce additional sets of control variables. Across all models, the coefficient on the core explanatory variable, LTCI, remains positive and statistically significant at the 1% level. This indicates that LTCI significantly enhances the SWB of its rural beneficiaries. Furthermore, the magnitude of the LTCI coefficient shows minimal variation as more controls are added across the specifications. This stability confirms the robustness of our baseline model and its findings.

**Table 4:** Baseline regression results.

Variable	(1)	(2)	(3)	(4)	(5)
	SWB	SWB	SWB	SWB	SWB
LTCI	2.466***	1.367***	1.365***	1.385***	1.288***
	(9.35)	(4.20)	(4.21)	(4.15)	(3.98)
Gender			0.952	0.904	0.868
			(0.75)	(0.65)	(0.63)
Age			19.175*	16.119	14.239
			(1.68)	(1.32)	(1.24)
Edu			−0.286	−0.298	−0.279
			(−0.82)	(−0.85)	(−0.80)
Marry			1.214**	1.082**	1.097**
			(2.36)	(2.22)	(2.23)
Social			0.504***	0.492***	0.468***
			(2.87)	(2.97)	(2.87)
Retire			−0.057	−0.053	−0.157
			(−0.13)	(−0.12)	(−0.37)
Exercise			0.360	0.400	0.430
			(0.93)	(1.02)	(1.09)
Drink_smoke				0.395	0.410
				(1.14)	(1.16)
Sleep				1.285**	1.302**
				(2.17)	(2.17)
Income_total				4.315*	3.846
				(1.72)	(1.50)
Family_size				0.047	0.046
				(0.52)	(0.52)
Fcamt				0.033	0.034
				(1.09)	(1.12)
Ins				−0.024	−0.040
				(−0.06)	(−0.11)
Pension				0.229	0.251
				(1.43)	(1.54)
Health_inst					−0.540
					(−1.58)
Hospitals					−0.061
					(−0.15)
Beds					−1.397
					(−1.16)
Gdp					−0.713
					(−0.86)
Old					0.148
					(1.06)
Year	Yes	Yes	Yes	Yes	Yes
ID	Yes	Yes	Yes	Yes	Yes
N	5,849	5,849	5,849	5,849	5,849
R-squared	0.014	0.013	0.021	0.030	0.034

T-values in parentheses. ∗*p* < 0.1, ∗∗*p* < 0.05, ∗∗∗*p* < 0.01. SEs clustered at city level. Year = time fixed effects, id = individual fixed effects (hereafter).

### 5.2 Parallel trend test

**Figure 3:**
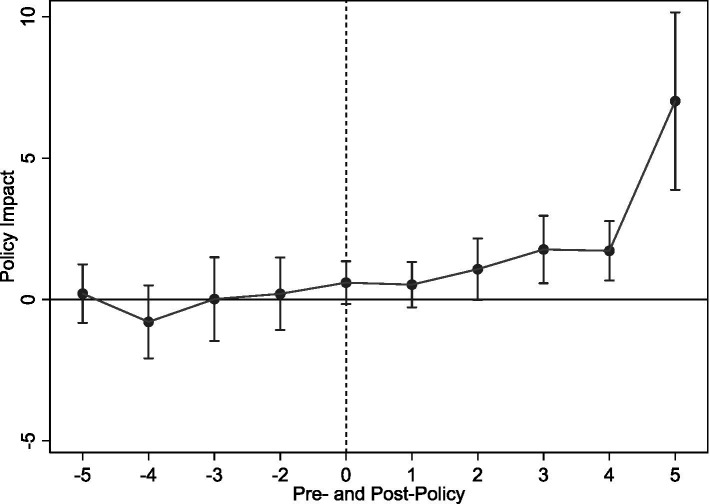
Parallel trend test.

Figure 3 presents parallel trend test results, using the year pre-policy implementation as baseline. Findings show statistically insignificant pre-implementation coefficients, suggesting no significant pre-LTCI pilot differences between pilot and non-pilot cities. Thus, the parallel trend assumption holds. Furthermore, coefficients become significantly positive 2 years post-implementation, indicating LTCI, despite a lag, positively impacts rural middle-aged and older SWB.

### 5.3 Placebo test

#### 5.3.1 Temporal placebo test

To address the concern that our findings are driven by pre-existing time trends rather than the policy itself, we conduct a temporal placebo test. This test assesses whether significant differences in SWB existed between the treatment and control groups of rural middle-aged and older individuals prior to the actual policy change. Specifically, we fictitiously advance the official LTCI implementation dates by two, three, and 4 years, respectively. The baseline model from Equation 1 is then re-estimated for each of these hypothetical timings. The results of these placebo tests are presented in Table 5.

**Table 5:** Temporal placebo test.

Variable	(1)	(2)	(3)
	SWB	SWB	SWB
LTCI	0.273	0.229	0.244
	(0.98)	(0.81)	(0.87)
Control	YES	YES	YES
Year	YES	YES	YES
ID	YES	YES	YES
N	5,849	5,849	5,849
R-squared	0.028	0.028	0.028

The estimated coefficients for these placebo treatments are all statistically insignificant, even at the 10% significance level. This suggests that there were no pre-existing systematic differences in the time trends of SWB between the treatment and control groups. This test, therefore, further corroborates the conclusion that LTCI indeed enhanced the SWB of rural middle-aged and older individuals, rather than the effect being driven by confounding temporal factors.

#### 5.3.2 City placebo test

**Figure 4:**
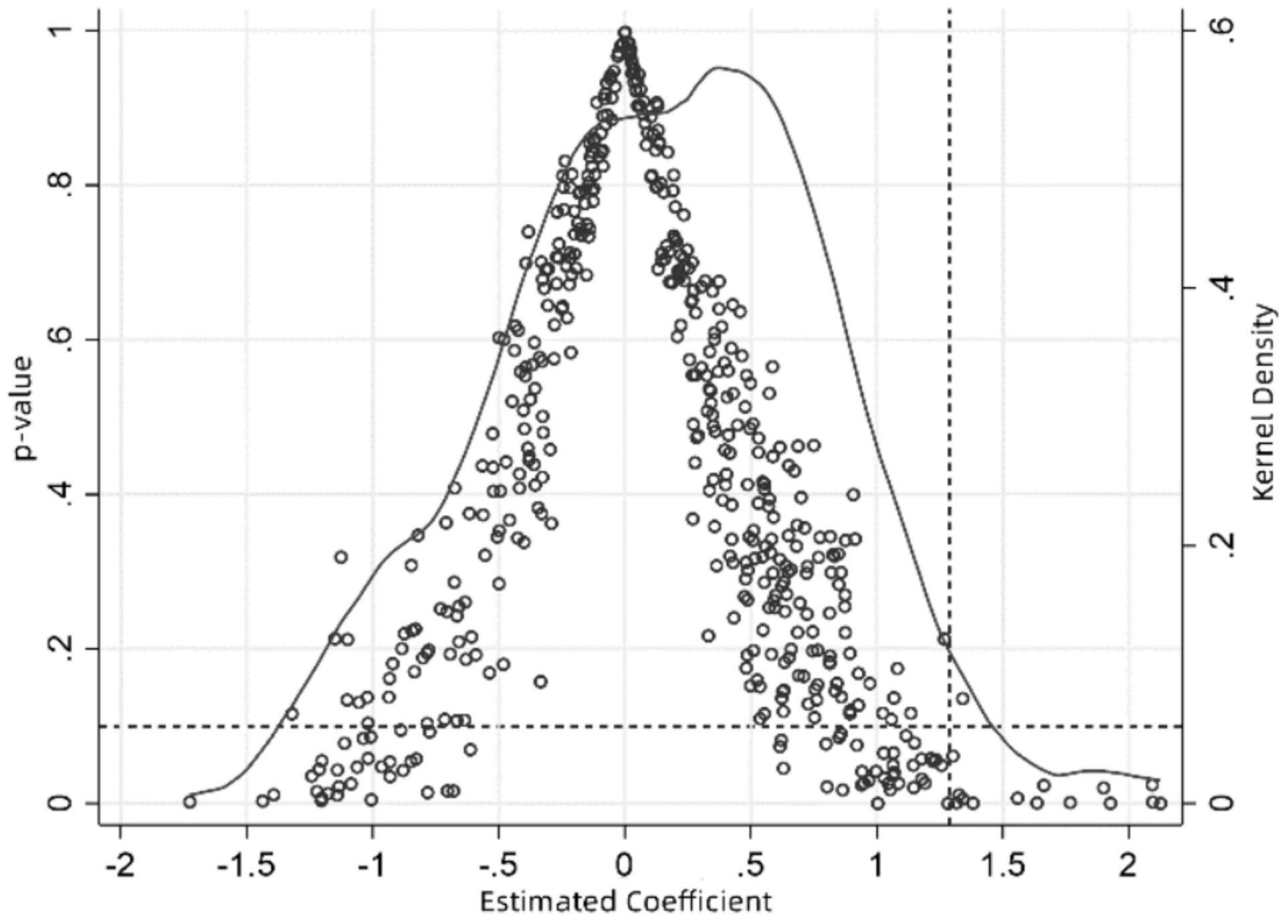
City-level robustness test. X-axis: Estimated coefficients from 500 random sample fictitious treatments. Hollow circles: *p*-values of placebo estimates. Solid curve: Kernel density of estimated coefficients. Right vertical dashed line: Actual baseline regression coefficient. Horizontal dashed line: *p* = 0.1.

To address potential omitted variable bias, this study conducts a city-level placebo test by randomly replacing treatment group cities. Procedure: First, 22 cities are randomly selected from the full sample as a fictitious treatment group; remaining cities form the control group. Then, LTCI’s effect on rural middle-aged and older SWB is estimated. Second, this process is repeated 500 times, yielding regression coefficients and *p*-values, whose distribution is shown in Figure 4. Placebo regression coefficients are normally distributed, cluster around zero, and are mostly statistically insignificant (10% level). In contrast, the actual estimated coefficient (1.288) is a significant outlier in this distribution (Figure 4, vertical dashed line). This suggests obtaining such an estimate under the null hypothesis is an extremely low probability, rare event. Therefore, unobserved factors are unlikely to drive this study’s baseline regression results.

### 5.4 Robustness tests

Although the preceding analyses have employed Propensity Score Matching (PSM) to balance observable covariates and placebo tests (Section 5.3) to provide initial evidence for the validity of our research design, a primary concern for causal inference remains the potential for bias from unobserved confounding variables and endogeneity. Therefore, to more rigorously test our baseline findings against these potential threats, this section presents an additional and comprehensive series of robustness and sensitivity checks. These tests are specifically designed to further address issues such as sample selection bias, violations of the parallel trends assumption, and sensitivity to alternative model specifications and measures.

#### 5.4.1 Propensity score matching difference-in-differences (PSM-DID) model

**Figure 5:**
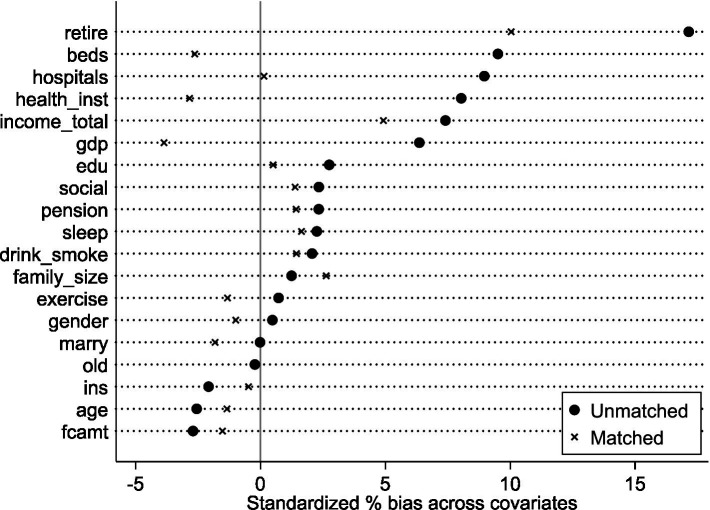
Balance test (1:3 matching).

**Figure 6:**
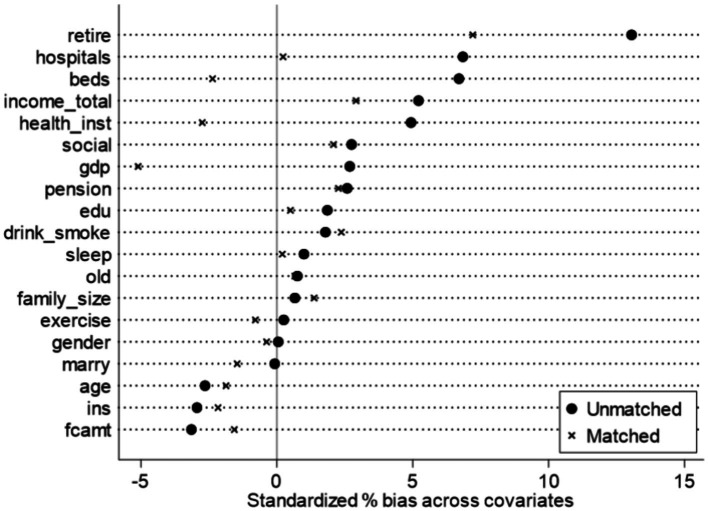
Balance test (1:2 matching).

**Figure 7:**
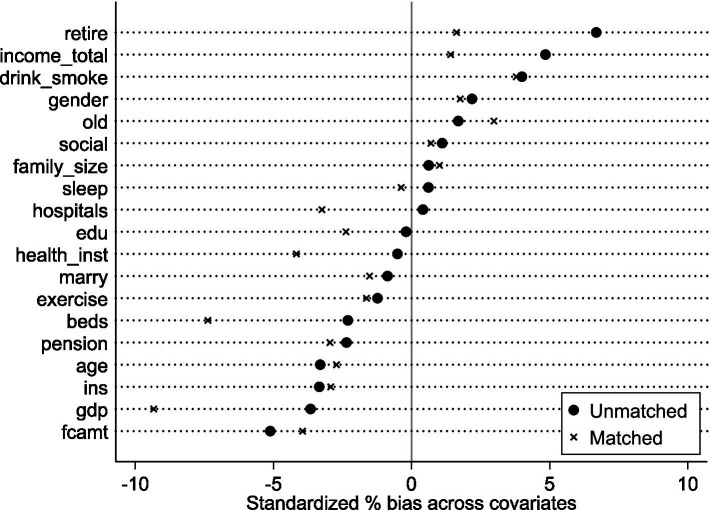
Balance test (1:1 matching).

This addresses endogeneity from reverse causality and sample selection bias. First, LTCI pilot implementation is unlikely influenced by middle-aged and older SWB, minimizing reverse causality concerns. Second, to mitigate sample selection bias, PSM uses nearest-neighbor matching (1:3, 1:2, 1:1 ratios). Post-matching balance tests show most covariates have no significant differences, satisfying the balance requirement. Matched sample regression results are in Table 6, Columns (1)–(3). Estimated coefficients remain statistically significant (5% level) across specifications, confirming LTCI indeed improves rural middle-aged and older SWB (Figures 5–7).

**Table 6:** Robustness test results (I).

Variable	(1)	(2)	(3)
	1:3	1:2	1:1
LTCI	1.444***	1.375***	1.631***
	(4.09)	(3.36)	(3.01)
Control	YES	YES	YES
Year	YES	YES	YES
ID	YES	YES	YES
N	4,909	3,857	2,630
R-squared	0.041	0.033	0.048

The caliper width is set at 0.05.

#### 5.4.2 Multi-period difference-in-differences and heterogeneous treatment effects

Recent literature highlights potential issues with heterogeneous treatment effects in multi-period DID models (32). Specifically, later-treated units may use earlier-treated units as controls, introducing bias from “bad controls” in traditional two-way fixed effects models. To examine this bias, we apply the Goodman-Bacon decomposition, categorizing estimates into three groups (Table 7). The “not-yet-treated” control group has a 0.03% weight. The “bad controls” (earlier-treated group) have a weight of only 0.02%. The “good controls” (never-treated group) have a positive coefficient and a dominant 95.9% weight. Results suggest “bad controls” have a negligible influence, while “good controls” drive the findings, confirming the robustness of baseline estimates.

**Table 7:** Goodman-bacon decomposition weights.

Group	Treated group	Control group	Weight	SWB
1	Earlier	Later	0.003	0.193
2	Later	Earlier	0.002	0.899
3	T	Never treated	0.959	1.250

Furthermore, we test the robustness of our findings using two alternative estimators designed for contexts with staggered treatment adoption. First, we employ the imputation estimator (33), which uses both never-treated and not-yet-treated samples to construct counterfactuals. Second, we use the stacking estimator (34), which identifies appropriate controls for each treated group before stacking the data by relative event time. As shown in Table 8, both of these advanced difference-in-differences estimators yield significantly positive results, confirming that our baseline findings are robust to alternative model specifications.

**Table 8:** Robust estimates under heterogeneous treatment effects.

Variable	(1)	(2)
LTCI	1.260***	0.669*
	(3.81)	(1.87)
N	5,652	9,489

#### 5.4.3 Parallel trend sensitivity test

**Figure 8:**
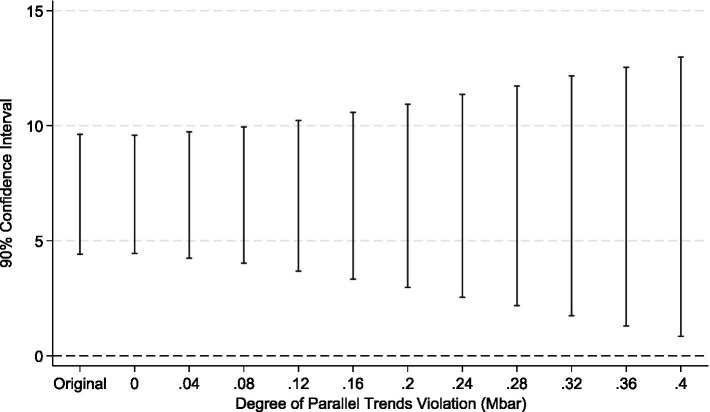
Relative deviation restriction.

**Figure 9:**
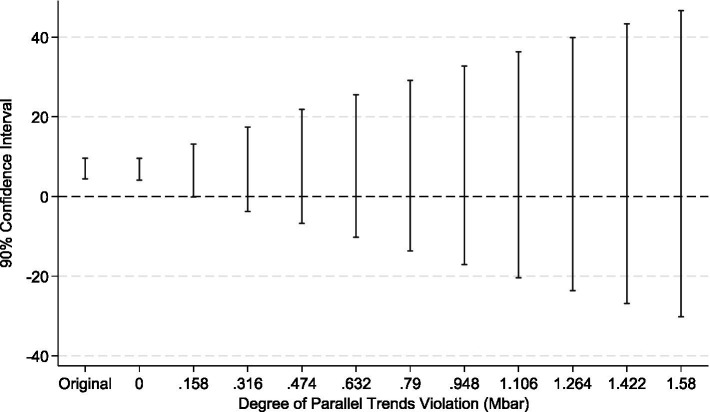
Smoothness restriction.

However, the theoretical literature on the Difference-in-Differences (DID) method indicates that conventional pre-treatment trend tests, due to their low statistical power, do not serve as valid evidence for the parallel trends assumption. This can even lead to biased estimations and inferences (35). To address this limitation, econometricians have proposed a counterfactual-based sensitivity analysis framework (36). The core of this method is to quantify the potential degree of deviation from the parallel trends assumption, denoted as *mbar*, and to test whether the treatment effect estimates remain robust under such a deviation. Therefore, this paper, drawing upon the research framework of Biasi and Sarsons (37), conducts a sensitivity analysis on the parallel trends assumption for the LTCI policy pilot. In the specific implementation, we define the shock matrix as matrix1 = (0\0\0\0\0\0\1) to verify the sensitivity of the policy’s impact on SWB after its effective date. The test results (see Figures 8, 9) reveal that after imposing the relative deviation and smoothness restrictions based on *mbar*, the parallel trends assumption remains robust. This suggests that even if the parallel trends assumption deviates to some extent, the positive effect of the LTCI policy on the SWB of middle-aged and older adults in rural China remains significant and reliable.

#### 5.4.4 Further robustness checks

To further validate the robustness of our findings, we conducted a series of additional tests. First, to mitigate potential endogeneity from unobservable confounders, we refined the control group by selecting cities that had not implemented LTCI by 2020 but were designated as new pilots post-2020, ensuring greater comparability with the original treatment group. Second, we employed the Reweighted Semi-parametric DID (Abadie SDID) model, which maintains valid inference even if the strict parallel trends assumption is not fully met.

Third, we tested the robustness of our results using alternative dependent variables and additional controls. We used positive and negative emotions as proxy indicators for SWB. The positive emotions metric is based on two survey items (“hopeful,” “very happy”), while the negative emotions metric is constructed from six items (“annoyed by trivia,” “depressed,” “difficult to do anything,” “fearful,” “lonely,” and “unable to continue life”). Additionally, to account for potential bias from the COVID-19 pandemic, we introduced controls for pandemic-related factors, specifically infection of a close family member and days spent in quarantine or self-isolation.

**Figure 10:**
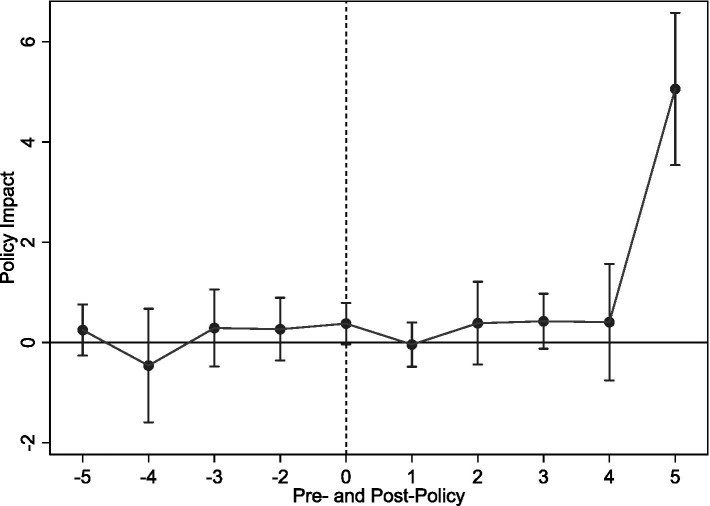
Parallel trend test (2).

**Figure 11:**
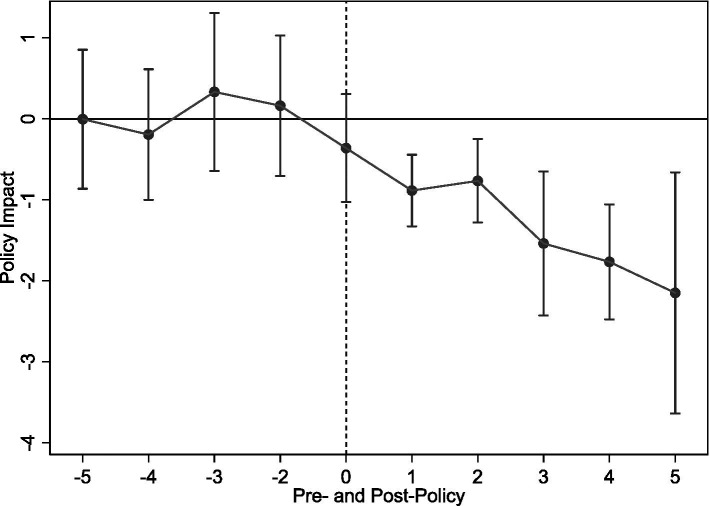
Parallel trend test (3).

The results for all these checks, presented in Table 9, are highly consistent with our baseline conclusion. The effect of LTCI remains statistically significant across all specifications: with the refined control group (Column 1), using the alternative econometric model (Column 2), with the alternative dependent variables (Columns 3-4), and after including the additional pandemic-related controls (Columns 5-7). The corresponding parallel trend tests for the alternative dependent variables (Figures 10, 11) also support this conclusion, underscoring the robustness of our core finding.

**Table 9:** Robustness test results (II).

Variable	(1)	(2)	(3)	(4)	(5)	(6)	(7)
	SWB	SWB	PA	NA	SWB	SWB	SWB
LTCI	1.389**	1.946***	0.390**	−1.127***	1.294***	1.294***	1.296***
	(2.54)	(10.51)	(2.10)	(−5.25)	(4.00)	(3.98)	(4.01)
Control	Yes	Yes	Yes	Yes	Yes	Yes	Yes
Year	Yes	Yes	Yes	Yes	Yes	Yes	Yes
ID	Yes	Yes	Yes	Yes	Yes	Yes	Yes
N	1,433	4,653	5,842	5,849	5,849	5,849	5,849
R-squared	0.066	-	0.021	0.038	0.034	0.034	0.034

## 6 Further analysis

### 6.1 Examination of mechanisms

Baseline results confirm LTCI significantly enhances rural middle-aged and older SWB. But through which pathways does LTCI exert this positive effect, and do actual effects align with our proposed theories? Following Jiang (38), this study examines three potential channels: medical burden, health status, and consumption level.

#### 6.1.1 Medical burden

Our theoretical framework suggests that LTCI improves well-being by alleviating the medical burden on rural middle-aged and older individuals. We test this mechanism using four proxy variables: out-of-pocket annual inpatient expenses post-reimbursement (MB1), out-of-pocket outpatient expenses (MB2), annual inpatient admissions (MB3), and annual outpatient visits (MB4). The results, presented in Table 10 (Columns 1-4), show that the LTCI coefficient is significantly negative for all four indicators. This confirms that the policy effectively reduces both inpatient and outpatient expenses and the frequency of hospital admissions and visits, thus clearly alleviating the medical burden for its beneficiaries.

**Table 10:** Mechanism test (I).

Variable	(1)	(2)	(3)	(4)
	MB1	MB2	MB3	MB4
LTCI	−0.453***	−0.219*	−0.121***	−0.394***
	(−2.69)	(−1.85)	(−2.72)	(−3.09)
Control	Yes	Yes	Yes	Yes
Year	Yes	Yes	Yes	Yes
ID	Yes	Yes	Yes	Yes
N	5,849	5,834	5,849	5,849
R-squared	0.063	0.065	0.014	0.020

Our theoretical analysis suggested relieving medical burden enhances SWB via multiple pathways. Extensive literature confirms reduced medical burden significantly improves well-being (17–19). These findings provide strong evidence that LTCI promotes rural middle-aged and older SWB by alleviating medical burden, thus confirming Hypothesis 1a.

#### 6.1.2 Health status

LTCI may indirectly enhance rural middle-aged and older SWB by improving health status. To test this, we use three health status proxies: (1) a composite score for difficulties with activities of daily living (ADL), including, but not limited to, housework, cooking, shopping, and managing finances (H1; lower = better); (2) self-rated health (H2; higher = better); and (3) presence of any of 14 chronic diseases (e.g., hypertension, diabetes) (H3). Table 11, Columns (1)–(3), reports LTCI’s estimated effects on these health indicators. All LTCI coefficients are statistically significant, indicating the policy effectively improves this population’s health outcomes.

**Table 11:** Mechanism test (II).

Variable	(1)	(2)	(3)	(4)	(5)
	H1	H2	H3	TC	EC
LTCI	−0.094**	0.165***	−0.238***	0.028**	0.024**
	(−2.46)	(2.66)	(−6.23)	(2.08)	(2.07)
Control	Yes	Yes	Yes	Yes	Yes
Year	Yes	Yes	Yes	Yes	Yes
ID	Yes	Yes	Yes	Yes	Yes
N	5,744	5,555	5,849	5,679	5,779
R-squared	0.042	0.020	0.107	0.038	0.022

Extensive literature confirms better health significantly enhances SWB (20–22). These findings strongly suggest LTCI improves rural middle-aged and older SWB by promoting better health, thus confirming Hypothesis 2.

#### 6.1.3 Consumption level

Consistent with our theoretical framework, LTCI may also enhance the SWB of rural middle-aged and older individuals by stimulating consumption. We test this mechanism with two variables: logged household per capita consumption (TC), calculated as total household consumption divided by household size, and enjoyment-oriented consumption (EC), which includes spending on cultural and recreational activities, tourism, beauty services, and education. The results in Table 11 (Columns 4–5) show that the LTCI coefficient is statistically significant and positive for both consumption measures, confirming that the policy significantly promotes both overall and enjoyment-oriented consumption.

Consistent with theory, substantial research shows increased consumption effectively enhances SWB (26–28). These results strongly evidence that LTCI promotes rural middle-aged and older SWB by encouraging consumption, thus confirming Hypothesis 3.

### 6.2 Heterogeneity analysis

#### 6.2.1 Income level

To examine heterogeneity by income level, we conduct subgroup regressions for high- and low-income samples. The results in Table 12 (Columns 1–2) show that while the LTCI coefficient is significantly positive for both groups, the effect is substantially more pronounced for the low-income group. A possible explanation is that low-income groups, facing greater care needs with less economic security, derive more substantial well-being improvements from the financial and caregiving support that LTCI provides. In contrast, higher-income individuals may have access to other resources (e.g., family support or savings), resulting in smaller marginal benefits from the policy.

**Table 12:** Heterogeneity analysis.

Variable	(1)	(2)	(3)	(4)	(5)	(6)
	L-Inc.	H-Inc.	L-Hlth	H-Hlth	L-Edu.	H-Edu.
LTCI	3.154***	0.822**	1.850***	1.142**	1.639***	1.116***
	(2.88)	(2.18)	(3.90)	(2.57)	(4.00)	(3.45)
Control	Yes	Yes	Yes	Yes	Yes	Yes
Year	Yes	Yes	Yes	Yes	Yes	Yes
ID	Yes	Yes	Yes	Yes	Yes	Yes
N	2,908	2,941	4,267	1,582	4,157	1,692
R-squared	0.093	0.041	0.056	0.039	0.044	0.061
Coefficient of inter-group difference (*P*-value)	0.000	0.000	0.000	0.000	0.000	0.000

The *p*-value for the difference in coefficients between groups is calculated using a bootstrap-based Fisher’s permutation test with 1,000 replications.

#### 6.2.2 Health status

We further explore heterogeneity by health status, dividing the sample into healthy and unhealthy groups. The results in Table 12 (Columns 3-4) indicate that the policy’s positive impact is significantly more pronounced for individuals in poorer health. A plausible explanation is that these individuals have more urgent needs for insurance and therefore perceive greater protection and benefit from LTCI, leading to a more notable improvement in their SWB.

#### 6.2.3 Educational attainment

To analyze heterogeneity by education, we perform subgroup regressions for low- and high-education groups. The results (Table 12, Columns 5-6) show that the enhancing effect of LTCI on SWB is significantly stronger for individuals with lower educational attainment. A possible reason is that individuals with lower education may face greater economic and health pressures, making the care and financial support from LTCI particularly impactful. Furthermore, the policy might enhance their perception of social fairness and reduce health-related anxiety.

## 7 Conclusion and policy implications

Using CHARLS data and a multi-period DID model, this study investigates LTCI’s impact on rural middle-aged and older SWB and its underlying mechanisms. Empirical findings show: First, LTCI significantly improves rural middle-aged and older SWB. Second, mechanisms include reducing medical burdens, improving health, and stimulating consumption. Third, the positive SWB impact is more pronounced for low-income, less healthy, and lower-educated rural groups.

Based on these findings, the following policy recommendations are proposed:

First, expand LTCI coverage and inclusiveness. Given the significant SWB improvement for rural and disadvantaged populations (low-income, poor-health, low-education), we recommend expanding LTCI coverage, focusing on rural areas. Resources should be precisely allocated to high-risk groups (e.g., low-income, health-vulnerable). Additionally, payment schemes linked to rural disposable income (e.g., proportional or differentiated contributions) should be explored to alleviate financial burdens.
Second, the operational mechanisms of LTCI should be optimized to more effectively enhance SWB by strengthening the three core channels identified in this study. To further reduce medical burdens, policy should focus on increasing reimbursement rates, especially for chronic and major illnesses, and on strengthening the integration of urban and rural health insurance systems to ease economic pressure. To directly improve health outcomes, the program could expand to include more health management services, such as regular check-ups and chronic disease management, while grassroots organizations enhance health education to promote preventive care. Finally, to promote consumption that enriches quality of life, the government could use targeted subsidies to encourage older adult participation in cultural, recreational, and tourism activities.
Third, to holistically advance China’s social long-term care insurance (LTCI) system, a multi-tiered policy architecture is essential. From a macro-level perspective, the central government must establish a unified national legislative framework to standardize the system beyond its pilot stage. This involves clarifying sustainable financing mechanisms, likely through tripartite contributions from the state, employers, and individuals’ social insurance accounts, and defining core principles for eligibility and benefits nationwide to ensure equity. At the meso-level, municipal governments should be empowered to tailor implementation based on local economic conditions and demographic needs. Their focus must be on integrating health, older adult care, and social security resources, cultivating a regulated market of designated care providers, and establishing robust assessment and quality control mechanisms. At the micro-level, policy must concentrate on refining service delivery by strengthening community-based and home-based care networks, ensuring fair and transparent eligibility evaluations for individuals, and optimizing reimbursement protocols to guarantee accessible, affordable, and high-quality care for the insured.

## Data Availability

The original contributions presented in the study are included in the article/supplementary material, further inquiries can be directed to the corresponding author.

## Footnotes

^1^Some pilot cities were excluded because they were not included in the CHARLS database. Additionally, Shanghai was excluded to avoid potential confounding effects from its “Senior Citizen Medical Care Program” (2016–2018), a policy with features similar to LTCI. Beijing was removed because its Haidian District launched a similar policy in 2016, and the available data do not allow for district-level distinctions.
